# Dichloridobis(1,3-phenyl­propane-1,3-dionato-κ^2^ *O*,*O*′)tin(IV) toluene hemisolvate

**DOI:** 10.1107/S1600536810028084

**Published:** 2010-07-21

**Authors:** Chun Keng Thy, Kong Mun Lo, Seik Weng Ng

**Affiliations:** a Department of Chemistry, University of Malaya, 50603 Kuala Lumpur, Malaysia

## Abstract

The two Sn—O—C—C—C—O chelate rings in the title compound, [Sn(C_15_H_11_O_2_)_2_Cl_2_]·0.5C_7_H_8_, adopt envelope conformations, with the Sn atom deviating from the least-squares plane passing through the C and O atoms by 0.626 (1) Å in one ring and by 0.690 (1) Å for the other. The two planes are aligned at an angle of 59.6 (1)°. The Cl atoms occupy *cis* positions in the octa­hedral SnCl_2_O_4_ coordination environment. The solvent mol­ecule is disordered about a center of inversion.

## Related literature

For the crystal structure of anhydrous dichloro­dibis(1,3-phenyl­propane-1,3-dionato)tin(IV), see: Searle *et al.* (1989 ▶).
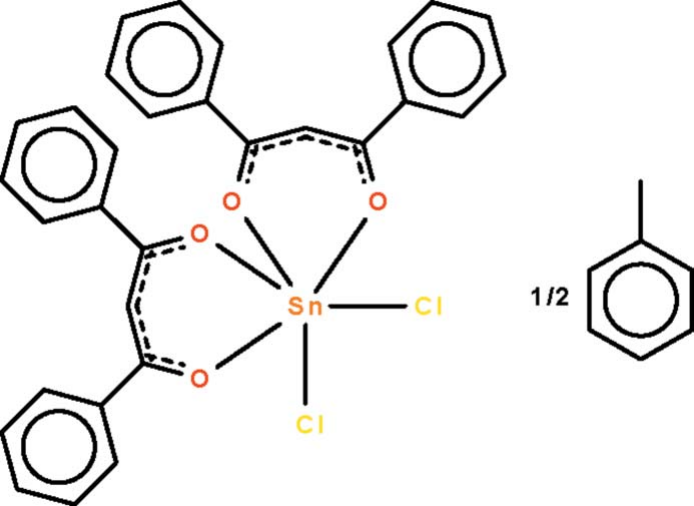


## Experimental

### 

#### Crystal data

[Sn(C_15_H_11_O_2_)_2_Cl_2_]·0.5C_7_H_8_
*M* *_r_* = 682.13
Monoclinic, 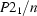

*a* = 8.0024 (1) Å
*b* = 21.5554 (2) Å
*c* = 16.6838 (2) Å
β = 97.2740 (5)°
*V* = 2854.71 (6) Å^3^
*Z* = 4
Mo *K*α radiation
μ = 1.12 mm^−1^
*T* = 100 K
0.40 × 0.35 × 0.30 mm

#### Data collection

Bruker SMART APEX diffractometer
Absorption correction: multi-scan (*SADABS*; Sheldrick, 1996 ▶) *T*_min_ = 0.663, *T*_max_ = 0.730
23143 measured reflections
6534 independent reflections
6269 reflections with *I* > 2σ(*I*)
*R*_int_ = 0.014

#### Refinement

*R*[*F* ^2^ > 2σ(*F* ^2^)] = 0.018
*wR*(*F* ^2^) = 0.049
*S* = 1.02
6534 reflections
386 parameters
43 restraints
H-atom parameters constrained
Δρ_max_ = 0.51 e Å^−3^
Δρ_min_ = −0.48 e Å^−3^

### 

Data collection: *APEX2* (Bruker, 2009 ▶); cell refinement: *SAINT* (Bruker, 2009 ▶); data reduction: *SAINT*; program(s) used to solve structure: *SHELXS97* (Sheldrick, 2008 ▶); program(s) used to refine structure: *SHELXL97* (Sheldrick, 2008 ▶); molecular graphics: *X-SEED* (Barbour, 2001 ▶); software used to prepare material for publication: *publCIF* (Westrip, 2010 ▶).

## Supplementary Material

Crystal structure: contains datablocks global, I. DOI: 10.1107/S1600536810028084/bt5299sup1.cif

Structure factors: contains datablocks I. DOI: 10.1107/S1600536810028084/bt5299Isup2.hkl

Additional supplementary materials:  crystallographic information; 3D view; checkCIF report

## Figures and Tables

**Fig. 1.**
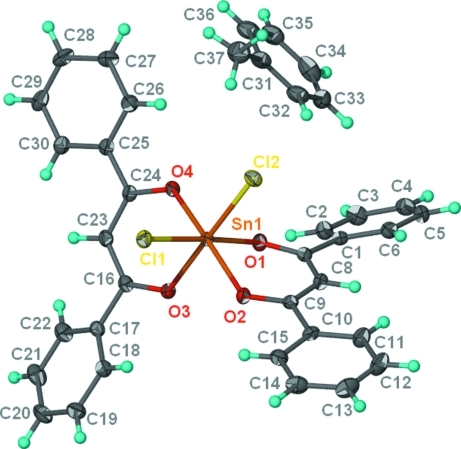
Anisotropic displacement ellipsoid plot (Barbour, 2001) of SnCl2(C15H11O2).0.5C7H8 at the 70% probability level. Hydrogen atoms are drawn as spheres of arbitrary radius.

## supplementary crystallographic information

### Comment

The toluene solvate (Scheme I) was obtained from the reaction of dibenzyltin dichloride and dibenzoylmethane in ethanol. Since no toluene was used in the synthesis, the toluene in the crystal structure would have resulted from the cleavage of the tin–carbon_benzyl_ bond by the dibenzoylmethane molecule, which in the deprotonated form chelates to tin (Fig. 1). The six-membered Sn–O–C–C–C–O chelate rings adopt an envelope-shaped conformation, with the tin atom lying off the least-squares plane defined by the carbon/oxygen atoms. Bond dimensions are similar to those reported for the anhydrous compound (Searle *et al.*, 1989).

### Experimental

Dibenzyltin dichloride (1 mmol) and dibenzoylmethane (1 mmol) were refluxed in ethanol for 1 h. The toluene solvate was obtained as crystals when the solvent was allowed to evaporate.

### Refinement

The toluene molecule is disordered over a center-of-inversion. The aromatic ring was refined as a hexagon of 1.39 Å sides, and the C_methyl_–C_phenyl_ distance was refined with a distance restraint of 1.54±0.01 Å; the displacement parameters of the seven carbon atoms were restrained to be nearly isotropic.

Hydrogen atoms were placed in calculated positions (C–H 0.95–0.98 Å) and included in the refinement in the riding model approximation, with *U*(H) set to 1.2–1.5*U*_eq_(C).

### Crystal data

**Table:**

[Sn(C_15_H_11_O_2_)_2_Cl_2_]·0.5C_7_H_8_	*F*(000) = 1372
*M**_r_* = 682.13	*D*_x_ = 1.587 Mg m^−^^3^
Monoclinic, *P*2_1_/*n*	Mo *K*α radiation, λ = 0.71073 Å
Hall symbol: -P 2yn	Cell parameters from 9775 reflections
*a* = 8.0024 (1) Å	θ = 2.6–28.3°
*b* = 21.5554 (2) Å	µ = 1.12 mm^−^^1^
*c* = 16.6838 (2) Å	*T* = 100 K
β = 97.2740 (5)°	Block, colorless
*V* = 2854.71 (6) Å^3^	0.40 × 0.35 × 0.30 mm
*Z* = 4	

### Data collection

**Table:**

Bruker SMART APEX diffractometer	6534 independent reflections
Radiation source: fine-focus sealed tube	6269 reflections with *I* > 2σ(*I*)
graphite	*R*_int_ = 0.014
ω scans	θ_max_ = 27.5°, θ_min_ = 1.6°
Absorption correction: multi-scan (*SADABS*; Sheldrick, 1996)	*h* = −10→10
*T*_min_ = 0.663, *T*_max_ = 0.730	*k* = −28→27
23143 measured reflections	*l* = −21→20

### Refinement

**Table:**

Refinement on *F*^2^	Primary atom site location: structure-invariant direct methods
Least-squares matrix: full	Secondary atom site location: difference Fourier map
*R*[*F*^2^ > 2σ(*F*^2^)] = 0.018	Hydrogen site location: inferred from neighbouring sites
*wR*(*F*^2^) = 0.049	H-atom parameters constrained
*S* = 1.02	*w* = 1/[σ^2^(*F*_o_^2^) + (0.0264*P*)^2^ + 1.8695*P*] where *P* = (*F*_o_^2^ + 2*F*_c_^2^)/3
6534 reflections	(Δ/σ)_max_ = 0.001
386 parameters	Δρ_max_ = 0.51 e Å^−^^3^
43 restraints	Δρ_min_ = −0.48 e Å^−^^3^

### Fractional atomic coordinates and isotropic or equivalent isotropic displacement parameters (Å^2^)

**Table:**

	*x*	*y*	*z*	*U*_iso_*/*U*_eq_	Occ. (<1)
Sn1	0.423028 (11)	0.234239 (4)	0.547401 (5)	0.01263 (4)	
Cl1	0.20470 (4)	0.170342 (15)	0.48111 (2)	0.01801 (7)	
Cl2	0.23181 (4)	0.308712 (15)	0.58666 (2)	0.01822 (7)	
O1	0.62198 (12)	0.28585 (4)	0.60498 (6)	0.01569 (18)	
O2	0.43258 (12)	0.18534 (4)	0.65338 (6)	0.01634 (19)	
O3	0.59905 (12)	0.17245 (4)	0.51420 (6)	0.01660 (19)	
O4	0.45921 (13)	0.28244 (4)	0.44524 (6)	0.01604 (19)	
C1	0.74806 (17)	0.35237 (6)	0.70667 (8)	0.0146 (2)	
C2	0.87260 (18)	0.37017 (7)	0.65982 (9)	0.0187 (3)	
H2	0.8871	0.3476	0.6123	0.022*	
C3	0.97545 (18)	0.42080 (7)	0.68234 (9)	0.0219 (3)	
H3	1.0615	0.4321	0.6508	0.026*	
C4	0.95274 (18)	0.45488 (7)	0.75081 (9)	0.0209 (3)	
H4	1.0223	0.4898	0.7658	0.025*	
C5	0.82797 (19)	0.43778 (7)	0.79729 (9)	0.0199 (3)	
H5	0.8114	0.4613	0.8438	0.024*	
C6	0.72730 (18)	0.38632 (6)	0.77598 (8)	0.0176 (3)	
H6	0.6440	0.3742	0.8087	0.021*	
C7	0.63927 (16)	0.29790 (6)	0.68135 (8)	0.0142 (2)	
C8	0.56859 (18)	0.26349 (6)	0.73948 (8)	0.0160 (3)	
H8	0.5858	0.2782	0.7936	0.019*	
C9	0.47456 (16)	0.20909 (6)	0.72458 (8)	0.0142 (2)	
C10	0.41507 (17)	0.17367 (6)	0.79168 (8)	0.0153 (2)	
C11	0.42603 (19)	0.19764 (7)	0.87008 (9)	0.0208 (3)	
H11	0.4811	0.2361	0.8825	0.025*	
C12	0.3567 (2)	0.16530 (8)	0.92983 (9)	0.0257 (3)	
H12	0.3618	0.1823	0.9826	0.031*	
C13	0.2798 (2)	0.10820 (8)	0.91288 (10)	0.0259 (3)	
H13	0.2328	0.0862	0.9540	0.031*	
C14	0.27184 (19)	0.08334 (7)	0.83542 (10)	0.0230 (3)	
H14	0.2213	0.0439	0.8239	0.028*	
C15	0.33758 (18)	0.11603 (6)	0.77509 (9)	0.0183 (3)	
H15	0.3300	0.0992	0.7221	0.022*	
C16	0.62450 (16)	0.16168 (6)	0.44027 (8)	0.0146 (2)	
C17	0.70704 (16)	0.10122 (6)	0.42799 (8)	0.0153 (3)	
C18	0.67625 (18)	0.05155 (6)	0.47798 (9)	0.0182 (3)	
H18	0.6084	0.0576	0.5201	0.022*	
C19	0.74452 (19)	−0.00663 (7)	0.46630 (9)	0.0225 (3)	
H19	0.7217	−0.0404	0.4998	0.027*	
C20	0.84583 (19)	−0.01515 (7)	0.40570 (10)	0.0243 (3)	
H20	0.8925	−0.0549	0.3978	0.029*	
C21	0.87930 (19)	0.03398 (7)	0.35662 (10)	0.0245 (3)	
H21	0.9500	0.0280	0.3156	0.029*	
C22	0.80949 (18)	0.09223 (7)	0.36718 (9)	0.0202 (3)	
H22	0.8316	0.1257	0.3331	0.024*	
C23	0.57756 (17)	0.20051 (6)	0.37482 (8)	0.0158 (3)	
H23	0.5989	0.1865	0.3231	0.019*	
C24	0.50103 (17)	0.25886 (6)	0.37916 (8)	0.0140 (2)	
C25	0.45972 (16)	0.29810 (6)	0.30672 (8)	0.0145 (2)	
C26	0.36654 (18)	0.35218 (6)	0.31416 (9)	0.0187 (3)	
H26	0.3362	0.3637	0.3653	0.022*	
C27	0.31824 (19)	0.38901 (7)	0.24702 (9)	0.0221 (3)	
H27	0.2554	0.4258	0.2523	0.027*	
C28	0.36168 (19)	0.37208 (7)	0.17210 (9)	0.0222 (3)	
H28	0.3264	0.3969	0.1260	0.027*	
C29	0.45660 (19)	0.31897 (7)	0.16435 (9)	0.0211 (3)	
H29	0.4874	0.3078	0.1131	0.025*	
C30	0.50646 (17)	0.28211 (7)	0.23134 (8)	0.0171 (3)	
H30	0.5723	0.2460	0.2260	0.020*	
C31	0.3563 (8)	0.4768 (3)	0.4741 (4)	0.0210 (10)	0.50
C32	0.4464 (8)	0.4664 (3)	0.5497 (4)	0.0265 (13)	0.50
H32	0.4019	0.4398	0.5872	0.032*	0.50
C33	0.6015 (8)	0.4950 (3)	0.5706 (4)	0.0285 (14)	0.50
H33	0.6631	0.4879	0.6223	0.034*	0.50
C34	0.6666 (8)	0.5339 (3)	0.5158 (5)	0.0315 (15)	0.50
H34	0.7726	0.5535	0.5300	0.038*	0.50
C35	0.5765 (9)	0.5443 (4)	0.4401 (5)	0.0266 (12)	0.50
H35	0.6210	0.5709	0.4027	0.032*	0.50
C36	0.4214 (9)	0.5158 (3)	0.4193 (4)	0.0260 (12)	0.50
H36	0.3599	0.5229	0.3676	0.031*	0.50
C37	0.1816 (4)	0.44999 (16)	0.4561 (2)	0.0292 (7)	0.50
H37A	0.1831	0.4064	0.4730	0.044*	0.50
H37B	0.1441	0.4527	0.3980	0.044*	0.50
H37C	0.1041	0.4734	0.4857	0.044*	0.50

### Atomic displacement parameters (Å^2^)

**Table:**

	*U*^11^	*U*^22^	*U*^33^	*U*^12^	*U*^13^	*U*^23^
Sn1	0.01623 (5)	0.01190 (5)	0.00992 (5)	0.00085 (3)	0.00233 (3)	−0.00018 (3)
Cl1	0.02183 (16)	0.01610 (15)	0.01564 (15)	−0.00272 (11)	0.00060 (12)	−0.00137 (11)
Cl2	0.01985 (15)	0.01574 (15)	0.01997 (16)	0.00295 (11)	0.00608 (12)	−0.00062 (12)
O1	0.0185 (5)	0.0165 (5)	0.0126 (4)	−0.0016 (4)	0.0038 (4)	−0.0010 (4)
O2	0.0221 (5)	0.0148 (4)	0.0119 (4)	−0.0004 (4)	0.0012 (4)	−0.0007 (3)
O3	0.0206 (5)	0.0164 (5)	0.0128 (4)	0.0043 (4)	0.0021 (4)	−0.0008 (4)
O4	0.0231 (5)	0.0134 (4)	0.0122 (4)	0.0012 (4)	0.0048 (4)	0.0003 (3)
C1	0.0153 (6)	0.0138 (6)	0.0143 (6)	0.0014 (5)	0.0007 (5)	0.0015 (5)
C2	0.0188 (6)	0.0195 (7)	0.0181 (7)	0.0000 (5)	0.0040 (5)	−0.0013 (5)
C3	0.0184 (7)	0.0219 (7)	0.0260 (8)	−0.0028 (5)	0.0060 (6)	0.0015 (6)
C4	0.0186 (7)	0.0165 (6)	0.0264 (7)	−0.0016 (5)	−0.0020 (6)	0.0007 (5)
C5	0.0249 (7)	0.0169 (6)	0.0174 (7)	0.0007 (5)	0.0005 (5)	−0.0023 (5)
C6	0.0199 (6)	0.0171 (6)	0.0160 (6)	0.0005 (5)	0.0032 (5)	0.0010 (5)
C7	0.0140 (6)	0.0141 (6)	0.0144 (6)	0.0028 (5)	0.0013 (5)	−0.0007 (5)
C8	0.0183 (6)	0.0169 (7)	0.0126 (6)	0.0000 (5)	0.0015 (5)	−0.0005 (5)
C9	0.0148 (6)	0.0145 (6)	0.0135 (6)	0.0032 (5)	0.0021 (5)	0.0012 (5)
C10	0.0150 (6)	0.0165 (6)	0.0143 (6)	0.0016 (5)	0.0012 (5)	0.0032 (5)
C11	0.0232 (7)	0.0230 (7)	0.0161 (7)	−0.0050 (5)	0.0017 (5)	0.0014 (5)
C12	0.0297 (8)	0.0335 (8)	0.0140 (7)	−0.0048 (6)	0.0035 (6)	0.0032 (6)
C13	0.0277 (8)	0.0292 (8)	0.0217 (7)	−0.0021 (6)	0.0071 (6)	0.0106 (6)
C14	0.0239 (7)	0.0186 (7)	0.0271 (8)	−0.0015 (5)	0.0060 (6)	0.0057 (6)
C15	0.0207 (7)	0.0156 (6)	0.0191 (7)	0.0020 (5)	0.0040 (5)	0.0011 (5)
C16	0.0137 (6)	0.0141 (6)	0.0162 (6)	−0.0021 (5)	0.0027 (5)	−0.0021 (5)
C17	0.0142 (6)	0.0140 (6)	0.0171 (6)	0.0004 (5)	−0.0008 (5)	−0.0029 (5)
C18	0.0196 (6)	0.0168 (6)	0.0178 (7)	0.0010 (5)	0.0006 (5)	−0.0017 (5)
C19	0.0254 (7)	0.0153 (6)	0.0254 (7)	0.0014 (5)	−0.0018 (6)	−0.0002 (5)
C20	0.0206 (7)	0.0178 (7)	0.0330 (8)	0.0049 (5)	−0.0025 (6)	−0.0080 (6)
C21	0.0189 (7)	0.0249 (7)	0.0304 (8)	0.0029 (6)	0.0063 (6)	−0.0080 (6)
C22	0.0186 (7)	0.0190 (7)	0.0236 (7)	−0.0003 (5)	0.0049 (5)	−0.0028 (5)
C23	0.0196 (6)	0.0151 (6)	0.0133 (6)	−0.0001 (5)	0.0048 (5)	−0.0017 (5)
C24	0.0151 (6)	0.0138 (6)	0.0132 (6)	−0.0026 (5)	0.0025 (5)	−0.0006 (5)
C25	0.0155 (6)	0.0144 (6)	0.0138 (6)	−0.0032 (5)	0.0023 (5)	0.0002 (5)
C26	0.0218 (7)	0.0178 (7)	0.0168 (6)	0.0003 (5)	0.0036 (5)	0.0009 (5)
C27	0.0229 (7)	0.0185 (7)	0.0245 (7)	0.0020 (5)	0.0015 (6)	0.0044 (6)
C28	0.0217 (7)	0.0248 (7)	0.0188 (7)	−0.0041 (6)	−0.0023 (5)	0.0081 (6)
C29	0.0231 (7)	0.0268 (7)	0.0134 (6)	−0.0057 (6)	0.0025 (5)	0.0018 (5)
C30	0.0180 (6)	0.0184 (6)	0.0150 (6)	−0.0029 (5)	0.0029 (5)	−0.0001 (5)
C31	0.023 (2)	0.018 (2)	0.024 (2)	0.0032 (17)	0.0075 (19)	0.0015 (16)
C32	0.041 (3)	0.017 (3)	0.025 (2)	0.006 (2)	0.017 (2)	0.0064 (19)
C33	0.034 (3)	0.028 (3)	0.021 (2)	0.011 (2)	−0.003 (2)	−0.004 (2)
C34	0.030 (2)	0.026 (3)	0.042 (3)	−0.0038 (19)	0.016 (2)	−0.010 (2)
C35	0.033 (2)	0.017 (2)	0.032 (3)	−0.0018 (16)	0.012 (2)	0.0031 (19)
C36	0.034 (2)	0.019 (2)	0.028 (2)	0.0038 (18)	0.0139 (19)	0.0056 (17)
C37	0.0278 (16)	0.0270 (16)	0.0324 (17)	−0.0008 (12)	0.0020 (13)	−0.0004 (13)

### Geometric parameters (Å, °)

**Table:**

Sn1—O4	2.0477 (9)	C17—C18	1.3981 (19)
Sn1—O2	2.0515 (10)	C18—C19	1.391 (2)
Sn1—O3	2.0646 (9)	C18—H18	0.9500
Sn1—O1	2.0756 (10)	C19—C20	1.386 (2)
Sn1—Cl2	2.3667 (3)	C19—H19	0.9500
Sn1—Cl1	2.3840 (3)	C20—C21	1.385 (2)
O1—C7	1.2904 (16)	C20—H20	0.9500
O2—C9	1.2981 (16)	C21—C22	1.395 (2)
O3—C16	1.2962 (16)	C21—H21	0.9500
O4—C24	1.2957 (16)	C22—H22	0.9500
C1—C6	1.3961 (19)	C23—C24	1.4049 (18)
C1—C2	1.3959 (19)	C23—H23	0.9500
C1—C7	1.4906 (18)	C24—C25	1.4779 (18)
C2—C3	1.390 (2)	C25—C26	1.3978 (19)
C2—H2	0.9500	C25—C30	1.3997 (19)
C3—C4	1.389 (2)	C26—C27	1.387 (2)
C3—H3	0.9500	C26—H26	0.9500
C4—C5	1.389 (2)	C27—C28	1.388 (2)
C4—H4	0.9500	C27—H27	0.9500
C5—C6	1.390 (2)	C28—C29	1.389 (2)
C5—H5	0.9500	C28—H28	0.9500
C6—H6	0.9500	C29—C30	1.388 (2)
C7—C8	1.3963 (19)	C29—H29	0.9500
C8—C9	1.3984 (19)	C30—H30	0.9500
C8—H8	0.9500	C31—C32	1.3900
C9—C10	1.4826 (18)	C31—C36	1.3900
C10—C11	1.399 (2)	C31—C37	1.507 (6)
C10—C15	1.4005 (19)	C32—C33	1.3900
C11—C12	1.388 (2)	C32—H32	0.9500
C11—H11	0.9500	C33—C34	1.3900
C12—C13	1.389 (2)	C33—H33	0.9500
C12—H12	0.9500	C34—C35	1.3900
C13—C14	1.393 (2)	C34—H34	0.9500
C13—H13	0.9500	C35—C36	1.3900
C14—C15	1.386 (2)	C35—H35	0.9500
C14—H14	0.9500	C36—H36	0.9500
C15—H15	0.9500	C37—H37A	0.9800
C16—C23	1.3893 (19)	C37—H37B	0.9800
C16—C17	1.4870 (18)	C37—H37C	0.9800
C17—C22	1.3962 (19)		
			
O4—Sn1—O2	169.82 (4)	O3—C16—C23	125.49 (12)
O4—Sn1—O3	86.03 (4)	O3—C16—C17	114.46 (12)
O2—Sn1—O3	87.00 (4)	C23—C16—C17	120.02 (12)
O4—Sn1—O1	86.12 (4)	C22—C17—C18	119.43 (13)
O2—Sn1—O1	86.20 (4)	C22—C17—C16	122.25 (12)
O3—Sn1—O1	87.74 (4)	C18—C17—C16	118.30 (12)
O4—Sn1—Cl2	92.91 (3)	C19—C18—C17	120.31 (13)
O2—Sn1—Cl2	93.72 (3)	C19—C18—H18	119.8
O3—Sn1—Cl2	177.21 (3)	C17—C18—H18	119.8
O1—Sn1—Cl2	89.61 (3)	C20—C19—C18	119.86 (14)
O4—Sn1—Cl1	94.56 (3)	C20—C19—H19	120.1
O2—Sn1—Cl1	92.76 (3)	C18—C19—H19	120.1
O3—Sn1—Cl1	89.29 (3)	C19—C20—C21	120.31 (14)
O1—Sn1—Cl1	176.90 (3)	C19—C20—H20	119.8
Cl2—Sn1—Cl1	93.366 (12)	C21—C20—H20	119.8
C7—O1—Sn1	123.02 (8)	C20—C21—C22	120.20 (14)
C9—O2—Sn1	124.27 (8)	C20—C21—H21	119.9
C16—O3—Sn1	124.44 (8)	C22—C21—H21	119.9
C24—O4—Sn1	126.03 (8)	C17—C22—C21	119.88 (14)
C6—C1—C2	119.22 (13)	C17—C22—H22	120.1
C6—C1—C7	121.41 (12)	C21—C22—H22	120.1
C2—C1—C7	119.37 (12)	C16—C23—C24	125.10 (12)
C3—C2—C1	120.31 (13)	C16—C23—H23	117.4
C3—C2—H2	119.8	C24—C23—H23	117.4
C1—C2—H2	119.8	O4—C24—C23	123.80 (12)
C4—C3—C2	120.20 (13)	O4—C24—C25	114.60 (11)
C4—C3—H3	119.9	C23—C24—C25	121.60 (12)
C2—C3—H3	119.9	C26—C25—C30	119.42 (13)
C5—C4—C3	119.78 (13)	C26—C25—C24	118.11 (12)
C5—C4—H4	120.1	C30—C25—C24	122.44 (12)
C3—C4—H4	120.1	C27—C26—C25	120.19 (13)
C4—C5—C6	120.20 (13)	C27—C26—H26	119.9
C4—C5—H5	119.9	C25—C26—H26	119.9
C6—C5—H5	119.9	C28—C27—C26	120.04 (14)
C5—C6—C1	120.27 (13)	C28—C27—H27	120.0
C5—C6—H6	119.9	C26—C27—H27	120.0
C1—C6—H6	119.9	C27—C28—C29	120.19 (13)
O1—C7—C8	125.22 (12)	C27—C28—H28	119.9
O1—C7—C1	115.09 (12)	C29—C28—H28	119.9
C8—C7—C1	119.67 (12)	C30—C29—C28	120.11 (14)
C7—C8—C9	125.32 (13)	C30—C29—H29	119.9
C7—C8—H8	117.3	C28—C29—H29	119.9
C9—C8—H8	117.3	C29—C30—C25	120.02 (13)
O2—C9—C8	124.37 (12)	C29—C30—H30	120.0
O2—C9—C10	114.66 (12)	C25—C30—H30	120.0
C8—C9—C10	120.97 (12)	C32—C31—C36	120.0
C11—C10—C15	119.09 (13)	C32—C31—C37	118.7 (3)
C11—C10—C9	121.86 (12)	C36—C31—C37	121.1 (3)
C15—C10—C9	118.96 (12)	C33—C32—C31	120.0
C12—C11—C10	120.17 (14)	C33—C32—H32	120.0
C12—C11—H11	119.9	C31—C32—H32	120.0
C10—C11—H11	119.9	C32—C33—C34	120.0
C13—C12—C11	120.38 (15)	C32—C33—H33	120.0
C13—C12—H12	119.8	C34—C33—H33	120.0
C11—C12—H12	119.8	C35—C34—C33	120.0
C12—C13—C14	119.80 (14)	C35—C34—H34	120.0
C12—C13—H13	120.1	C33—C34—H34	120.0
C14—C13—H13	120.1	C36—C35—C34	120.0
C15—C14—C13	120.07 (14)	C36—C35—H35	120.0
C15—C14—H14	120.0	C34—C35—H35	120.0
C13—C14—H14	120.0	C35—C36—C31	120.0
C14—C15—C10	120.46 (14)	C35—C36—H36	120.0
C14—C15—H15	119.8	C31—C36—H36	120.0
C10—C15—H15	119.8		
			
O4—Sn1—O1—C7	153.37 (10)	C11—C12—C13—C14	−0.2 (2)
O2—Sn1—O1—C7	−33.32 (10)	C12—C13—C14—C15	−1.2 (2)
O3—Sn1—O1—C7	−120.46 (10)	C13—C14—C15—C10	1.1 (2)
Cl2—Sn1—O1—C7	60.43 (10)	C11—C10—C15—C14	0.3 (2)
O4—Sn1—O2—C9	74.0 (2)	C9—C10—C15—C14	−176.28 (13)
O3—Sn1—O2—C9	120.85 (10)	Sn1—O3—C16—C23	19.78 (18)
O1—Sn1—O2—C9	32.92 (10)	Sn1—O3—C16—C17	−158.46 (9)
Cl2—Sn1—O2—C9	−56.45 (10)	O3—C16—C17—C22	−150.91 (13)
Cl1—Sn1—O2—C9	−150.00 (10)	C23—C16—C17—C22	30.7 (2)
O4—Sn1—O3—C16	−29.25 (10)	O3—C16—C17—C18	30.91 (17)
O2—Sn1—O3—C16	158.17 (11)	C23—C16—C17—C18	−147.44 (13)
O1—Sn1—O3—C16	−115.51 (10)	C22—C17—C18—C19	−1.2 (2)
Cl1—Sn1—O3—C16	65.37 (10)	C16—C17—C18—C19	177.04 (13)
O2—Sn1—O4—C24	76.8 (2)	C17—C18—C19—C20	1.1 (2)
O3—Sn1—O4—C24	29.95 (11)	C18—C19—C20—C21	−0.1 (2)
O1—Sn1—O4—C24	117.95 (11)	C19—C20—C21—C22	−0.8 (2)
Cl2—Sn1—O4—C24	−152.63 (11)	C18—C17—C22—C21	0.3 (2)
Cl1—Sn1—O4—C24	−59.02 (11)	C16—C17—C22—C21	−177.86 (13)
C6—C1—C2—C3	0.5 (2)	C20—C21—C22—C17	0.7 (2)
C7—C1—C2—C3	−179.98 (13)	O3—C16—C23—C24	2.7 (2)
C1—C2—C3—C4	−1.3 (2)	C17—C16—C23—C24	−179.11 (13)
C2—C3—C4—C5	0.7 (2)	Sn1—O4—C24—C23	−20.39 (19)
C3—C4—C5—C6	0.7 (2)	Sn1—O4—C24—C25	158.82 (9)
C4—C5—C6—C1	−1.6 (2)	C16—C23—C24—O4	−2.7 (2)
C2—C1—C6—C5	1.0 (2)	C16—C23—C24—C25	178.13 (12)
C7—C1—C6—C5	−178.56 (13)	O4—C24—C25—C26	−6.43 (18)
Sn1—O1—C7—C8	22.71 (18)	C23—C24—C25—C26	172.79 (13)
Sn1—O1—C7—C1	−158.92 (8)	O4—C24—C25—C30	175.33 (12)
C6—C1—C7—O1	153.74 (12)	C23—C24—C25—C30	−5.4 (2)
C2—C1—C7—O1	−25.77 (18)	C30—C25—C26—C27	1.2 (2)
C6—C1—C7—C8	−27.79 (19)	C24—C25—C26—C27	−177.09 (13)
C2—C1—C7—C8	152.70 (13)	C25—C26—C27—C28	0.3 (2)
O1—C7—C8—C9	3.1 (2)	C26—C27—C28—C29	−1.4 (2)
C1—C7—C8—C9	−175.23 (13)	C27—C28—C29—C30	0.9 (2)
Sn1—O2—C9—C8	−21.43 (18)	C28—C29—C30—C25	0.6 (2)
Sn1—O2—C9—C10	158.76 (9)	C26—C25—C30—C29	−1.7 (2)
C7—C8—C9—O2	−4.1 (2)	C24—C25—C30—C29	176.55 (13)
C7—C8—C9—C10	175.70 (13)	C36—C31—C32—C33	0.0
O2—C9—C10—C11	−169.73 (13)	C37—C31—C32—C33	−174.4 (4)
C8—C9—C10—C11	10.4 (2)	C31—C32—C33—C34	0.0
O2—C9—C10—C15	6.79 (18)	C32—C33—C34—C35	0.0
C8—C9—C10—C15	−173.03 (13)	C33—C34—C35—C36	0.0
C15—C10—C11—C12	−1.8 (2)	C34—C35—C36—C31	0.0
C9—C10—C11—C12	174.76 (14)	C32—C31—C36—C35	0.0
C10—C11—C12—C13	1.7 (2)	C37—C31—C36—C35	174.2 (4)
